# Prognostic Value of Pretreatment Albumin-to-Alkaline Phosphatase Ratio in Cancer: A Meta-Analysis

**DOI:** 10.1155/2020/6661097

**Published:** 2020-12-10

**Authors:** Hailun Xie, Lishuang Wei, Shuangyi Tang, Jialiang Gan

**Affiliations:** ^1^Department of Colorectal and Anal Surgery, The First Affiliated Hospital, Guangxi Medical University, Nanning, Guangxi, China; ^2^Geriatric Respiratory Disease Ward, The First Affiliated Hospital, Guangxi Medical University, Nanning, Guangxi, China; ^3^Department of Pharmacy, The First Affiliated Hospital, Guangxi Medical University, Nanning, Guangxi, China

## Abstract

**Background:**

Recently, it has been reported that the pretreatment albumin-to-alkaline phosphatase ratio (AAPR) is related to the prognosis of various cancers. The purpose of this systematic review and meta-analysis was to explore the prognostic value of pretreatment AAPR on clinical outcomes in cancer.

**Methods:**

PubMed, Web of Science, Cochrane Library, and Embase were systematically searched for relevant research before May 2020. Stata 12 was utilized to extract the data and the characteristics of each study and to generate a pooled hazard ratio (HR) and 95% confidence interval (CI) to assess the relationship between pretreatment AAPR and survival outcomes.

**Results:**

We included 16 eligible published articles involving 5,716 patients. We found that low pretreatment AAPR was associated with poor overall survival (HR = 2.12, 95% CI: 1.80–2.50, *P* < 0.001), cancer-specific survival (HR = 2.89, 95% CI: 1.46–5.71, *P* < 0.001), disease-free survival (HR = 1.91, 95% CI: 1.43–2.53, P < 0.001), and progression-free survival (HR = 1.93, 95% CI: 1.49–2.52, P < 0.001). However, there was no statistical relationship between pretreatment AAPR and recurrence-free survival, distant-metastasis-free survival, or locoregional relapse-free survival. The correlation between pretreatment AAPR and overall survival did not change significantly when possible confounders were stratified. The sensitivity analysis showed that this study was reliable.

**Conclusions:**

Low pretreatment AAPR was significantly associated with adverse clinical outcomes of cancer. Pretreatment AAPR could be a valuable noninvasive prognostic indicator for cancer.

## 1. Introduction

According to the World Health Organization, cancer-related deaths accounted for approximately one-sixth of total global deaths in 2015. The latest Global Cancer Statistics estimated 18.1 million new cancer cases and 9.6 million cancer deaths worldwide in 2018. Asia accounts for nearly half of new cancer cases and nearly 70% of cancer deaths. In China, the incidence and death rate of cancer ranks first in the world, with about 3.804 million new cases and 2.296 million deaths [1]. Cancer remains one of the leading causes of death worldwide and is a major obstacle to increasing life expectancy in every country in the 21st century [2]. Therefore, it is imperative to find more simple and convenient biological indicators to predict the prognosis of cancer, especially in the Asian population.

In recent years, a large number of studies have reported that inflammation and nutritional status play important roles in the onset, development, and therapeutic response of cancer and are significant factors affecting the clinical outcome of cancer patients [3–5]. In clinical practice, inflammatory and nutritional status is usually determined by routine blood and biochemical markers. Many indicators based on inflammation and nutritional status have been used to predict the prognosis of cancer, including the Glasgow prognostic score [6], modified Glasgow prognostic score [7], C-reactive protein–albumin ratio [8], and Geriatric Nutritional Risk Index [9]. Albumin-to-alkaline phosphatase ratio (AAPR), as a new inflammatory and nutrition-related indicator, has attracted increasing attention. Recent evidence confirms that pretreatment AAPR is associated with adverse outcomes in patients with various cancers, including hepatocellular carcinoma (HCC) [10], nasopharyngeal carcinoma (NPC) [11], non-small-cell lung cancer (NSCLC) [12], upper tract urothelial carcinoma (UTUC) [13], and breast cancer [14]. However, due to the differences in study design and sample size of these studies, there are still some contrary results. The association between pretreatment AAPR and cancer outcomes remains controversial [15]. The prognostic value of pretreatment AAPR in cancer has rarely been systematically investigated.

Therefore, it is necessary to evaluate the relationship between pretreatment AAPR and cancer outcomes based on available evidence. We performed a systematic review and meta-analysis to investigate the prognostic value of pretreatment AAPR in cancer patients.

## 2. Methods

### 2.1. Search Strategy and Study Selection

We performed a systematic literature search on PubMed, Web of Science, Cochrane Library, and Embase, with a cutoff date of May 10, 2020. The search strategy combined main keywords and free words and used a combination of the following search terms: (“Albumin-to-Alkaline phosphatase” OR “Albumin/Alkaline phosphatase” OR “ALB/ALP” OR “ALB-ALP” OR “AAPR”) AND (“neoplasms” OR “carcinoma” OR “leukemia” OR “lymphoma”). To avoid duplicate studies, we also examined all authors and organizations of the included articles and assessed the recruitment period and number of patients in each article. Additionally, we conducted a manual review of references to identify potentially relevant studies from the retrieved publications. The registration number in the international prospective register of systematic reviews (PROSPERO) was CRD42020206902.

### 2.2. Inclusion and Exclusion Criteria

Based on the PICOS criteria, we strictly screened the eligible studies for inclusion in the meta-analysis: (1) population—patients with cancer; (2) intervention—patients receiving surgery, chemoradiotherapy, or nonstandard treatment; (3) comparison—cancer patients with low AAPR and high AAPR; (4) outcomes—primary outcome: overall survival (OS), secondary outcome: disease-free survival (DFS), cancer-specific survival (CSS), distant-metastasis-free survival (DMFS), locoregional relapse-free survival (LRRFS), progression-free survival (PFS), and recurrence-free survival (RFS); and (5) study design—comparative cohort study (retrospective study or prospective study). The exclusion criteria were as follows: (1) population—research on patients without cancer; (2) intervention—research on the value of AAPR after treatment; (3) comparison—grouping for AAPR is ≥3 groups, or hazard ratio (HR) and 95% confidence interval (CI) could not be extracted; (4) outcomes—studies without primary or secondary results; and (5) study design—one-arm comparison studies and literature types were abstracts, letters, editorials, reviews, expert opinions, or case reports.

### 2.3. Data Extraction and Quality Assessment

Two investigators (Hailun Xie and Lishuang Wei) independently extracted the data and evaluated the quality of all eligible studies. The extracted basic information included the name of the first author, publication year, country, types of cancer, sample capacity, patient characteristics (age, male/female percentage), cutoff value of AAPR, treatment methods, outcome, follow-up time, and analysis method. The extraction of prognostic indicators included HR with corresponding 95% CI. If only the Kaplan–Meier curve provided prognostic outcomes, we used Engauge Digitizer 4.1 software to obtain the estimated HR using the Tierney method [16]. The quality of the included studies was evaluated referring to the Newcastle–Ottawa Scale (NOS) [17]. The NOS score ranged from 0 to 9 points, which included three aspects: patient selection (0–4 points), comparability (0–2 points), and outcome (0–3 points). Studies with NOS scores of ≥6 were considered as high quality.

### 2.4. Statistical Analysis

Data analysis was performed by Stata version 13.0 (STATA Corporation, College Station, TX, USA). A pooled HR with 95% CI was calculated. Cochran's *Q* test and Higgins *I*^2^ statistics were used to evaluate the heterogeneity among the studies. The random effects model was applied when there was significant heterogeneity (*I*^2^ > 50% or *P* < 0.10). Otherwise, the fixed effects model was applied. Subgroup analysis and meta-regression analysis were used to assess the sources of heterogeneity. Sensitivity analysis was used to evaluate the reliability of the results by recalculating the pooled HR with 95% CI after deleting one study at a time. Begg's funnel plot was used to assess publication bias. In this study, *P* < 0.05 was considered statistically significant.

## 3. Results

### 3.1. Study Characteristics

**Figure 1:**
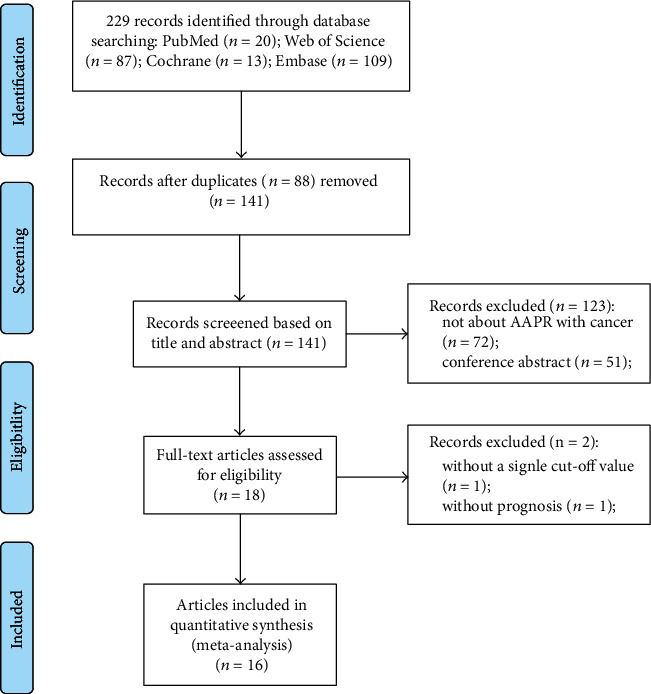
The flow chart of the literature selection.

A total of 229 published articles were initially retrieved based on the PRISMA guidelines. After deleting duplicate articles and unrelated literature, 141 articles required further evaluation. After the title and abstract screening, 123 articles were excluded for the following reasons: not about AAPR with cancer (*n* = 72) and conference abstracts (*n* = 51). We downloaded 18 full-text articles to further evaluate whether they could be included in our meta-analysis. One article was excluded because it did not have a single cutoff value, and another was excluded because it did not report prognosis. Finally, 16 articles involving 5,716 cases were included in our meta-analysis [10, 12–15, 18–27]. The flow chart of document retrieval is illustrated in Figure 1. Among the included articles, 15 were from China and one from South Korea. All included studies were retrospectively designed. Sixteen articles contained 20 cohort studies, among which one contained three cohort studies, and two contained two cohort studies. The publication year was between 2017 and 2020. The sample capacity ranged from 61 to 692, and the cutoff of AAPR varied from 0.35 to 0.68. This study involved a variety of cancers, including NPC, HCC, UTUC, breast cancer, NSCLC, lung cancer, SCLC, cholangiocarcinoma (CCA), cervical carcinoma, and extensive-disease SCLC (ED-SCLC). In terms of the quality evaluation of the selected cohort studies, the NOS score of 16 cohort studies was 8, the NOS score of one cohort study was 7, and the NOS score of three cohort studies was 6. The baseline information is shown in Table 1.

### 3.2. Association between Pretreatment AAPR and OS

**Figure 2:**
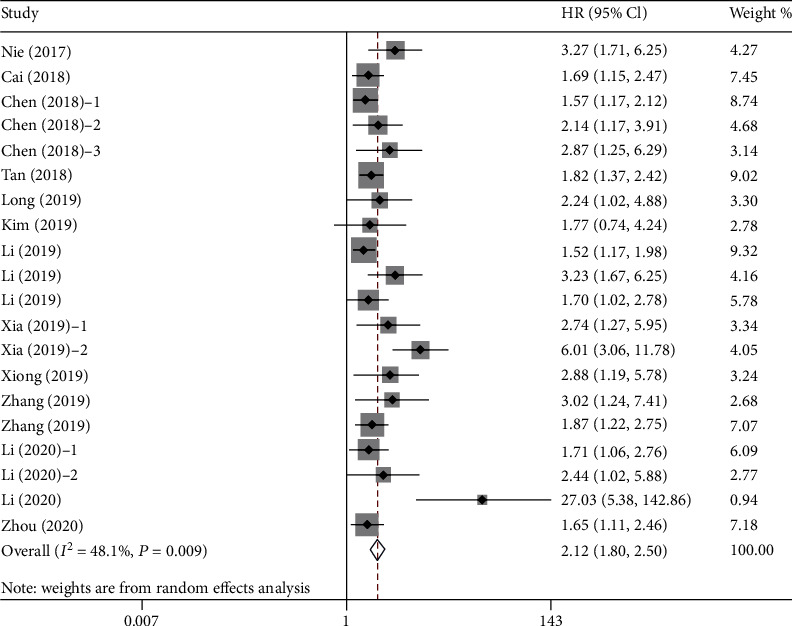
Forest plot for the association between pretreatment AAPR and OS. Abbreviations: OS: overall survival; AAPR: albumin-to-alkaline phosphatase ratio.

Twenty cohort studies enrolling 5,716 cases reported the prognostic significance of pretreatment AAPR for OS in cancer. Comprehensive results indicated that low pretreatment AAPR was significantly associated with poor OS, compared with high pretreatment AAPR (HR = 2.12, 95%CI = 1.80–2.50, *P* < 0.001). A random effects model was applied due to the obvious heterogeneity (*I*^2^ = 48.1%, *P* = 0.009) (Figure 2). The meta-analysis for OS included more than 10 studies, and heterogeneity was found in the results. Therefore, we conducted a subgroup analysis of publication year, country, sample capacity, cutoff value, cancer system, primary therapy, and analytic methods (Table 2). The results still indicated that low pretreatment AAPR had an adverse effect on OS in cancer patients. At the same time, when classified by these factors, heterogeneity was eliminated in some subgroup meta-analyses, such as publication year < 2019, sample capacity ≥ 230, cutoff value ≥ 0.5, respiratory cancer, chemotherapy, and mixed therapy subgroups. To explore further the impact of different subgroups on pooled HR, we conducted a meta-regression analysis. The influence of these different subgroups on pooled HR was not significant (*P*_study design_ = 0.763, *P*_country_ = 0.854, *P*_sample capacity_ = 0.608, *P*_cutoff value_ = 0.608, *P*_cancer system_ = 0.744, *P*_primary therapy_ = 0.114, and *P*_analytic method_ = 0.433).

### 3.3. Sensitivity Analysis for OS

**Figure 3:**
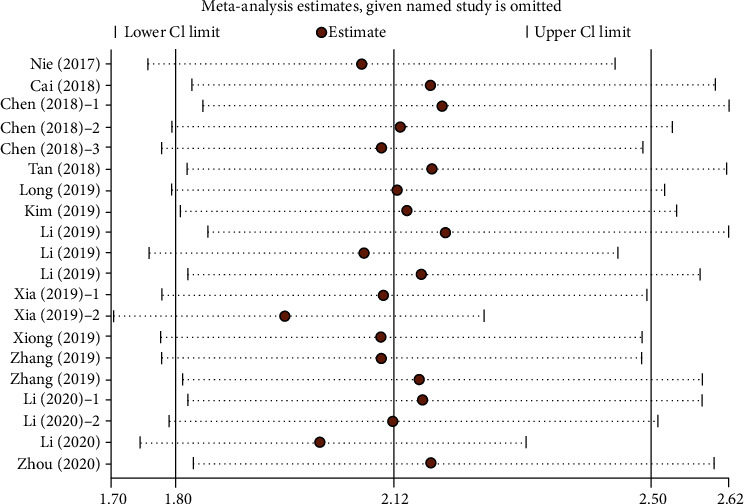
Sensitivity analysis for the association between pretreatment AAPR and OS. OS: overall survival.

Sensitivity analysis was used to assess the potential impact of the individual studies on the comprehensive results. We performed a sensitivity analysis by recalculating the pooled HR with 95% CI after deleting one study at a time (Figure 3). The results showed that omitting any included studies did not change the effect of pretreatment AAPR on the comprehensive meta-analysis of OS. In other words, the comprehensive result of our meta-analysis was stable.

### 3.4. Publication Bias for OS

**Figure 4:**
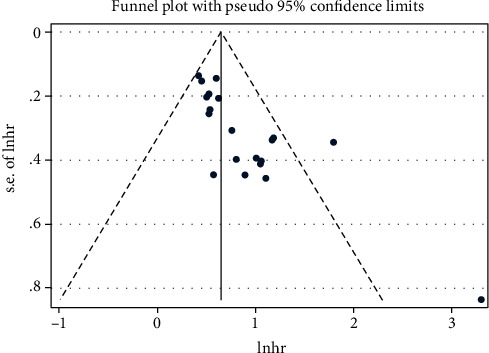
Begg's funnel plot for the assessment of potential publication bias according to OS. Abbreviations: OS: overall survival.

Begg's funnel plots were used to evaluate the potential publication bias (Figure 4). The funnel plot showed that the included studies presented asymmetry and the *P* value was < 0.05, suggesting that there was potential publication bias in the meta-analysis for OS.

### 3.5. Association between Pretreatment AAPR and Other Outcomes

**Figure 5:**
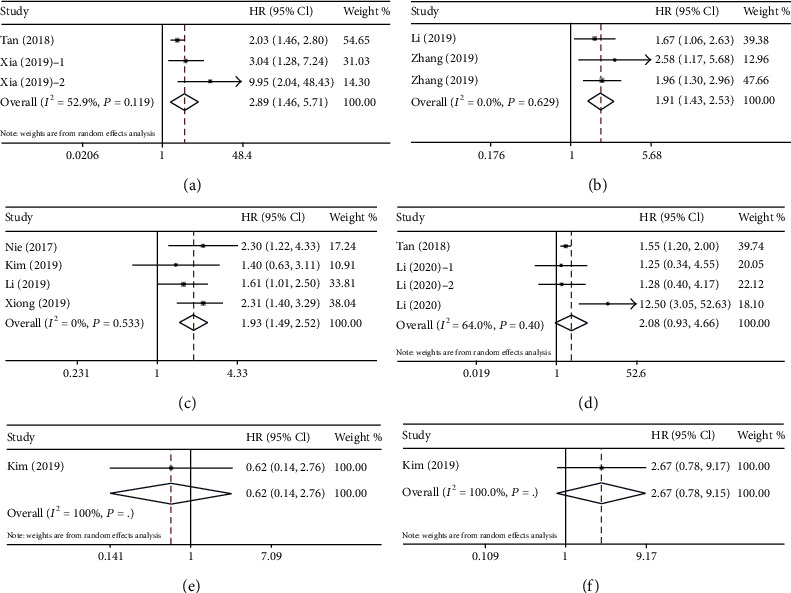
Forest plot for the association between pretreatment AAPR and other outcomes. (a) Forest plot for CSS. (b) Forest plot for DFS. (c) Forest plot for PFS. (d) Forest plot for RFS. (e) Forest plot for DMFS. (f) Forest plot for LRRFS. Abbreviations: CSS: cancer-specific survival; DFS: disease-free survival; PFS: progression-free survival; RFS: recurrence-free survival; DMFS: distant metastasis-free survival (DMFS); and LRRFS: locoregional relapse-free survival (LRRFS).

We studied the prognostic effect of pretreatment AAPR on CSS, DFS, PFS, RFS, DMFS, and LRRFS of cancer patients (Figure 5). Three cohort studies including 1,315 cases reported the prognostic value of pretreatment AAPR for CSS (Figure 5(a)). A random effects model (*I*^2^ = 52.9%, *P* = 0.119) was used because of the obvious heterogeneity. The combined results showed that patients with low pretreatment AAPR had poorer CSS than those with high pretreatment AAPR (HR = 2.89, 95% CI: 1.46–5.71, *P* < 0.001). Three cohort studies consisted of 1,116 patients reported HR for DFS (Figure 5(b)). The heterogeneity test indicated that there was no obvious heterogeneity, and the fixed effects model was adopted (*I*^2^ = 0.0%, *P* = 0.629). The pooled HR with 95% CI was 1.91 (95% CI: 1.43–2.53, *P* < 0.001), indicating a significant relationship between low pretreatment AAPR levels and poor DFS in cancer. Four cohort studies including 734 cases reported the prognostic value of pretreatment AAPR for PFS (Figure 5(c)). A fixed effects model (*I*^2^ = 0.0%, *P* = 0.533) was used. The results showed that patients with low pretreatment AAPR had worse PFS compared to patients with high pretreatment AAPR (HR = 1.93, 95% CI: 1.49–2.52, *P* < 0.001). However, there was no statistical relationship between pretreatment AAPR and RFS (HR = 2.08, 95% CI: 0.93–4.66, *P* = 0.076) (Figure 5(d)), DMFS (HR = 0.62, 95% CI: 0.14–2.76, *P* = 0.534) (Figure 5(e)), or LRRFS (HR = 2.67, 95% CI: 0.78–9.15, *P* = 0.117) (Figure 5(f)).

## 4. Discussion

AAPR, composed of albumin and alkaline phosphatase, was initially reported to be associated with poor prognosis of patients with HCC in 2015 [28]. Since then, many studies have demonstrated that pretreatment AAPR is a useful prognostic indicator for a variety of cancers. Pretreatment AAPR is expected to be a simple and effective tool for predicting the clinical outcome of cancer patients. However, the underlying mechanism of how pretreatment AAPR affects the prognosis of cancer remains unclear. As a composite indicator based on albumin and alkaline phosphatase, the prognostic value of pretreatment AAPR in cancer could be elucidated by investigating the function of its components (albumin and alkaline phosphatase). Albumin is a functional serum protein. It is an important clinical indicator that reflects the nutritional status and liver synthesis ability, and it has also been proved to regulate the inflammatory response throughout the body and play an antioxidant role in tumorigenesis [29]. Additionally, low albumin may affect the metabolism and function of immune cells, which may reduce immune function and cause adverse anticancer reactions [30]. Recently, many studies have shown that albumin is a useful prognostic predictor in various malignancies such as UTUC [31], HCC [32], and prostate cancer [33]. Alkaline phosphatase, a hydrolytic enzyme involved in biological processes, such as epithelial mesenchymal transformation and ERK1/2 dephosphorylation [34, 35], can cause the cessation of inflammatory signaling and induce an inhibitory immune response by regulating purinergic signaling. Alkaline phosphatase is mainly found in the liver, bone, and kidney. Alkaline phosphatase may be elevated in certain conditions like the liver, bone, and kidney diseases (malignant and benign) and especially in cholestasis. However, it is worth noting that Li et al. [15] showed that alkaline phosphatase has a pleiotropic effect in tumor progression, and it has prognostic value in cancer patients regardless of whether there is liver or bone metastasis. It has been confirmed to be elevated in various cancers and be associated with poor prognosis, including colorectal cancer, gastric cancer, and esophageal carcinoma [36–38]. Therefore, pretreatment with AAPR may be a more objective tumor marker that comprehensively reflects the balance between nutritional status and cancer-related inflammation.

Sixteen articles including 5,716 patients were included in our meta-analysis. In the meta-analysis of primary outcome, we found that cancer patients with low pretreatment AAPR had worse OS than those with high pretreatment AAPR. Stratified analysis was performed to correct for the influence of different subgroups on the results. The results showed that despite differences in publication year, sample capacity, cutoff value, cancer system, primary therapy, and analytical methods among different populations; the combined results still indicated that low pretreatment AAPR was correlated with poor OS. Further meta-regression analysis showed that the different subgroups did not affect the combined results of meta-analysis. We also conducted a sensitivity analysis by deleting one study at a time, and the results did not significantly change the pooled HR, indicating that our results were reliable. We also found a significant association between low pretreatment AAPR and adverse CSS, DFS and PFS in cancer patients. However, there was no statistical association between low pretreatment AAPR and adverse RFS, DMFS, and LRFRS. In summary, our meta-analysis confirmed that pretreatment AAPR can be a powerful predictor of poor outcomes in cancer patients. Although Guo et al. [39] conducted a meta-analysis on AAPR, our research carried out some new and useful explorations, mainly in the following aspects. Guo et al. mainly focused on the relationship between AAPR and OS. In our study, not only did we find that AAPR was associated with the primary outcome (OS) but we also found that AAPR was associated with poor secondary outcomes (CSS, DFS, and PFS), although there was no obvious correlation with outcomes such as RFS, DMFS, or LRFRS. We also conducted a more detailed subgroup analysis and meta-regression analysis to explore more comprehensively the value of AAPR in cancer patients. We also updated two studies including three cohorts. We also noted that there were some defects in the study of Guo et al.; for example, the research of Zhang et al. (2019) [24] on cervical carcinoma was not included and research by Chan et al. (2018) [28] showing that there were three categories of AAPR should be excluded. We believe that our study was more comprehensive and more accurate to summarize the relationship between AAPR and prognosis of cancer patients.

However, in view of some limitations, the results of our meta-analysis should be interpreted with caution. First, all included studies were conducted in Asia and were retrospective studies, which are more susceptible to potential selection bias. The current comprehensive conclusions mainly support the prognostic value of AAPR in Asian cancer patients. The practicality of AAPR is yet to be demonstrated in a prospective, multicenter study worldwide. Second, there was obvious heterogeneity in the meta-analysis of OS, but the meta-regression analysis showed that the subgroups were not the sources of the heterogeneity. We speculate that the sources of heterogeneity in this study might have been the small total sample size and the number of included studies, which need to be explored further in a large sample and multicenter prospective study. Third, there was publication bias in our study, but the sensitivity analysis showed that the results were reliable. Despite these limitations, we provided a meaningful exploration of the prognostic value of pretreatment AAPR in cancer patients based on available evidence.

## 5. Conclusion

This meta-analysis revealed that low pretreatment AAPR was significantly associated with adverse clinical outcomes in cancer patients. Pretreatment AAPR could be a valuable noninvasive prognostic indicator for cancer patients. Large, multicenter, prospective cohort studies are needed to evaluate further the role of pretreatment AAPR in cancer patients.

## Figures and Tables

**Table 1:** The characteristics of included studies.

Study (year)	Country	Cancer type	Sample capacity	Age (years)	Gender ratio	Treatment	Outcome	Follow-up (months)	Cutoff value	Analysis	NOS
Nie et al. [11]	China	Metastatic NPC	209	Median 45 (14-72)	179/30	With chemotherapy	OS, PFS	Median 16.6 (1-66.6)	0.447	M	8
Cai et al. [18]	China	Advanced HCC	237	Median 56 (45-66)	206/31	Without standard treatment	OS	>12	0.38	M	8
Chen et al. [10]	China	HCC	372	Median 52 (44-61)	348/24	With chemotherapy	OS	>60	0.439	M	8
			202	Median 56 (45-65)	176/26	With chemotherapy	OS	>60	0.439	M	8
			82	Median 55 (44-63)	73/9	With chemotherapy	OS	>60	0.439	M	8
Tan et al. [13]	China	UTUC	692	65.8 ± 11.4	398/294	With surgery	OS, CSS, RFS	Median 42 (20-75)	0.58	M	8
Long et al. [14]	China	Nonmetastatic breast cancer	746	NA	0/746	With surgery	OS	>60	0.525	M	8
Kim et al. [19]	Korea	Nonmetastatic NPC	100	NA	78/22	Mixed	OS, PFS, LRRFS, DMFS	Median 50.6	0.4876	M	8
Li et al. [15]	China	Metastatic NSCLC	290	Mean 55 (25-90)	210/75	With chemotherapy	OS	>60	0.84	M	8
Li et al. [20]	China	Lung cancer	390	63.0 ± 7.6	243/147	With surgery	OS, DFS	Median 50.0 (12-66)	0.57	M	8
Li et al. [21]	China	Limited stage SCLC	122	Median 58	244/58	With radiotherapy	OS, PFS	Median 21.3 (17.9-25.2)	0.61	U	6
Xia et al. [22]	China	Nonmetastatic RCC	419	61.0 ± 12.9	266/153	With surgery	OS, CSS	Median 50.0 (30.4-83.0)	0.39	M	8
			204	62.4 ± 11.7	127/77	With surgery	OS, CSS	Median 50.2 (29.8-83.1)	0.39	U	6
Xiong et al. [23]	China	CCA	303	Median 59 (29-83)	168/135	With surgery	OS, PFS	Median 21.0	0.41	M	8
Zhang et al. [24]	China	Cervical carcinoma	230	Median 55 (29-79)	101/65	With surgery	OS, DFS	Median 81 (12-137)	0.68	M	8
Zhang et al. [12]	China	NSCLC	496	Median 60 (34-81)	334/162	With surgery	OS, DFS	Median 47.0 (2.0-96.0)	0.64	M	8
Li et al. [25]	China	HCC	149	Mean 51.26	141/8	With surgery	OS, RFS	>60	0.38	U and M	7
			61	Mean 52.15	57/4	With surgery	OS, RFS	>60	0.38	U	6
Li et al. [26]	China	HCC	188	Median 52.3 (22-77)	161/27	With surgery	OS, RFS	Median 46.5	0.4	M	8
Zhou et al. [27]	China	ED-SCLC	224	60.51 ± 8.73	197/27	Mixed	OS	NA	0.35	M	8

**Table 2:** Stratification analysis for the meta-analysis with overall survival (OS) in patients with cancer.

Subgroup	No. of cohorts	No. of patients	Pooled HR (95% CI)	*P*	Heterogeneity	
					*I* ^2^ (%)	*P*_h_
Altogether	20	5716	2.12 (1.80-2.94)	<0.001	48.1	0.009
Publishing time						
<2019	6	1794	1.86 (1.55-2.23)	<0.001	11.9	0.339
≥2019	14	3922	2.30 (1.80-2.94)	<0.001	57.3	0.004
Country						
China	19	5616	2.14 (1.80-2.54)	<0.001	50.8	0.006
Korea	1	100	1.77 (0.74-4.24)	NA	NA	NA
Sample capacity						
<230	10	1541	2.52 (1.80-3.53)	<0.001	62.2	0.005
≥230	11	4175	1.81 (1.58-2.07)	<0.001	5.7	0.388
Cutoff value						
<0.5	14	3040	2.22 (1.76-2.81)	<0.001	60.2	0.002
≥0.5	6	2676	1.97 (1.63-2.38)	<0.001	0	0.575
Cancer system						
Digestive cancer	8	1594	2.13 (1.57-2.87)	<0.001	52.2	0.041
Respiratory cancer	7	1831	1.86 (1.51-2.28)	<0.001	25.6	0.234
Urinary cancer	3	1315	2.98 (1.41-6.32)	0.004	81	0.005
Breast cancer	1	746	2.24 (1.03-4.88)	NA	NA	NA
Gynecological cancers	1	230	3.02 (1.23-7.40)	NA	NA	NA
Primary therapy						
With chemotherapy	5	1155	1.88 (1.44-2.45)	<0.001	42.6	0.137
With radiotherapy	1	122	1.70 (1.03-2.80)	NA	NA	NA
With surgery	11	3878	2.64 (1.97-3.54)	<0.001	57.3	0.009
Mixed	2	324	1.67 (1.16-2.40)	0.006	0	0.889
Without treatment	1	237	1.69 (1.16-2.47)	NA	NA	NA
Analytic method						
Univariate	3	387	2.88 (1.28-6.48)	0.011	77.2	0.013
Multivariate	17	5329	1.99 (1.70-2.32)	<0.001	35.4	0.074

## Data Availability

Please contact author for data requests.

## Supplementary Materials

Supplementary Materials Supplemental files 1 PRISMA 2009 checklist.
